# Retraction Note: Oxytocin versus a combination of tranexamic acid and ethamsylate in reducing intraoperative bleeding during abdominal myomectomy: a randomized clinical trial

**DOI:** 10.1186/s12905-024-03419-y

**Published:** 2024-10-29

**Authors:** Ahmed Mahmoud Abdou, Elsayed Eldesouky, Elsayed Farag, Attia Mohammed, Doaa Fathy Mohamed Abdelaziz, A. Shaaban, Mostafa Ellaban, Abd Elhalim Mohamed Abd Elhalim, Ahmed Gamal Abo Elsror, Alrefaai Abd Elfattah Marai, Faiza Abdel-Hakam, Mohamed Abd-ElGawad, Asmaa Ahmed Elrashedy, Hanaa Abdelmonem, Mohamed Abdelmonem Kamel, Ibtesam K. Afiffi, Hazem Galal Abdelhameed Elsayed, Sameh Abdelmoneim Abdelhamed, Almandouh H. Bosilah, Heba Marie

**Affiliations:** 1 Obstetrics and Gynecology, Faculty of Medicine, Zagazig University, Zagazig, Egypt; 2 Department of Obstetrics and Gynecology, Faculty of Medicine, Alazhar University in Cairo, Cairo, Egypt; 3 Department of Obstetrics and Gynecology, Alazhar University for Girls in Cairo, Cairo, Egypt; 4 Department of Obstetrics and Gynecology, Faculty of Medicine, Alazhar University in Assuit, Assuit, Egypt; 5 International Islamic Institute for Studies and Population Research Alazhar University in Cairo, Cairo, Egypt; 6 MBBCh, Faculty of Medicine Fayoum University, Fayoum, Egypt; 7 Faculty of Medicine, Kafr El-Shaikh University, Kafr El-Shaikh, Egypt; 8 Department of Medical Microbiology and Immunology, Faculty of Medicine, Tanta University, Tanta, Egypt; 9 Department of Basic & Clinical Oral Sciences &, Faculty of Dental Medicine, Umm Al-Qura University, Makkah, Saudi Arabia; 10 Department of Obstetrics and Gynecology, Faculty of Medicine, Fayoum University, Fayoum, Egypt; 11 Department of Obstetrics and Gynecology, Faculty of Medicine, Damiata University, Damiata, Egypt; 12 Department of Obstetrics and Gynecology, Faculty of Medicine, Cairo University, Cairo, Egypt

**Retraction Note: BMC Women’s Health (2023) 23:398**

10.1186/s12905-023-02549-z

The Editor is retracting this article at the request of the corresponding author. After publication the authors were alerted to concerns with the data presented and they found that an error had occurred when the data had been copied to their analysis program from multiple sheets. Therefore the data analysed and presented in the article was not for the patients described in the study. The results and conclusions presented are therefore unreliable. Mohamed Abd-ElGawad, Asmaa Ahmed Elrashedy, Hanaa Abdelmonem and Mohamed Abdelmonem Kamel agree with this retraction. Elsayed Eldesouky, Elsayed Farag and Ibtesam K. Afiffi disagree with this retraction. Ahmed Mahmoud Abdou, Doaa Fathy Mohamed Abdelaziz, A. Shaaban, Mostafa Ellaban, Ahmed Gamal Abo Elsror, Alrefaai Abd Elfattah Marai, Faiza Abdel-Hakam, Hazem Galal Abdelhameed Elsayed, Sameh Abdelmoneim Abdelhamed and Heba Marie have not responded to correspondence from the Editor about this retraction. The Editor has not been able to find current email addresses for Attia Mohammed, Abd Elhalim Mohamed Abd Elhalim and Almandouh H. Bosilah.

